# Solitary necrotic nodule of the liver mimicking hepatocellular carcinoma: a case report

**DOI:** 10.1186/1757-1626-2-85

**Published:** 2009-01-25

**Authors:** Spiros G Delis, Dionysia A Kelgiorgi, Anastasios A Sofianidis, Charikleia C Triantopoulou, Ioannis A Papailiou, Christos G Dervenis

**Affiliations:** 1 Liver Surgical Unit, 1st Surgical Department, "Konstantopoulion" Hospital, Athens, Greece; 2 Department of Computed Tomography, "Konstantopoulion" Hospital, Athens, Greece

## Abstract

**Introduction:**

Solitary necrotic nodule of the liver is a rare lesion, with similar radiologic findings to those of hepatic metastases or other liver masses.

**Case presentation:**

We here report a case of a 30-year-old male with hepatic solitary necrotic nodule discovered after an episode of acute abdominal pain and high grade fever. Routine laboratory data revealed leukocytosis and abnormal liver function. The imaging features of the lesion suggested malignancy or liver adenoma. The patient underwent surgical resection of segments V and VI. Histology was compatible with solitary necrotic nodule and localized vein thrombosis at the periphery.

**Conclusion:**

Solitary necrotic nodule of the liver is a benign lesion which can mimic liver malignancies. Abdominal imaging and liver biopsy are often equivocal. In such circumstances liver resection is mandatory to exclude HCC or other malignant liver lesions.

## Case presentation

A 30-year-old Caucasian male with, no prior medical history, presented at the Emergency Department with acute right upper quadrant pain, high grade fever (38.5 C) and vomit.

Laboratory tests revealed marked leukocytosis (WBC: 19810/ml) and abnormal liver function tests (AST/ALT: 218/280 IU/L, ALP/γ-GT: 123/147 IU/L, TBil: O,84 mg/dl). Serology profile for hepatitis B or C was negative. A previous history of alcohol abuse was not documented.

Abdominal US revealed a 7 cm hyperechoic lesion at the right liver lobe.

**Figure 1:**
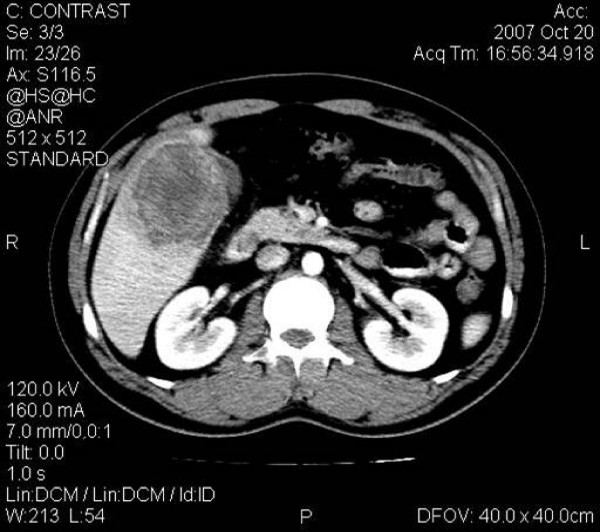
**Computed tomography image**. Computed tomography contrast-enhanced image, demonstrates a hemorrhagic lesion in liver segment V, presenting mild heterogeneous peripheral contrast enhancement.

Computed tomography demonstrated a single lesion, located in segments V and VI, with hemorrhagic features. After contrast administration the lesion appeared to be encapsulated with peripheral enhancement, a necrotic core and contrast wash out at the venous phase. These findings were suspicious for adenoma or HCC although fever and leukocytosis suggested a possible infectious process. No dilatation of the intra- or the extra-hepatic biliary ducts was noted. A lateral displacement of the gallbladder was documented (Figure 1).

The patient was admitted and initially treated with systemic broad spectrum antibiotics. However blood cultures and antibodies for Echinococcus and Entamoeba histolytica were negative.

Tumor markers including CA 19-9, a-Fetoprotein and CEA were within normal range.

A CT-guided core-needle biopsy was performed and revealed numerous lymphocytes and necrotic material but not atypia. Gram stain and Tissue cultures were negative.

The patient was treated with intravenous antibiotics for 7 days until fever subsides with complete white cell count normalization and followed by CT scan two weeks later. Due to high suspicion for a malignant process, anatomic liver resection including segments V and VI was performed. Intraoperatively, the liver had a normal appearance and a solid mass, well encapsulated without gallbladder invasion was identified.

Pathology was associated with hemorrhagic infarct and extensive hepatocellular necrosis, due to sinusoidal portal vein thrombosis. Furthermore hepatoportal sclerosis and non-cirrhotic portal vein fibrosis was identified. An eosinophil granular matter combined with calcifications, cholesterol, foam cells, and some inflammatory cells was noted.

The patient had an uneventful course, discharged the 6th postoperative day and remains well 6 months postoperatively.

## Discussion

Solitary necrotic nodule (SNN) of the liver is a rare benign lesion first reported in 1983 by Shepherd and Lee [1]. To date, fewer than 60 cases have been reported. There is a male predominance affected in the majority of cases the seventh and eighth decades of life. Most of the SNNs reported in the literature are benign and occurred in the right liver lobe.

In the majority of cases, this condition is clinically silent, and often the patients complain of nonspecific symptoms such as right upper quadrant pain. SNNs are usually first detected incidentally at US examination [2].

Lobular shape, well-delineated margin, and close proximity to the hepatic inflow structures, are suggestive of SNN, but are nondiagnostic. SNN usually appears as a well-defined lesion with no enhancement on contrast CT or MRI [3,4], although peripheral enhancement occurs occasionally on contrast MRI [3]. Radiological findings of SNN of the liver are similar to those of hepatic metastasis or other liver masses, such as hepatocellular carcinoma, pseudotumor, lymphoma, regenerative and dysplastic nodules, or some infectious processes [3]. Due to these similar radiological findings, making a correct differential diagnosis is often very difficult or impossible. The intraoperative US findings of the lesion also simulate hepatic malignancies, even though lesion multiplicity had been elucidated [3]. Furthermore, the cytological evaluation of specimens obtained by needle biopsy is not useful to correctly distinguish between these nodules and hepatic metastasis, because it often reveals the presence of necrotic tissue. Clinical symptoms when present as in our case very often misinterpret with inflammatory pseudotumor (IPT). As reported in literature, clinical and laboratory features of IPT of the liver also vary and are non specific. The most common symptom of liver IPT is abdominal pain, fever and weight loss [5,6]. The patients with IPT have leukocytosis and abnormal liver function tests with raise of serum CA 19-9. The imaging findings of hepatic IPT are also variable and non-specific and can present features that suggest hepatic malignancy (HCC or metastasis).

In our case reported, patient presented with a history of high grade fever and leukocytosis suggested an infectious process. CT findings were associated with a single lesion enhanced peripherally after contrast infusion with venous wash out. A necrotic center with intralesional bleeding was also identified. In this situation diagnosis of HCC or adenoma is possible and difficult to exclude even with needle core biopsy.

The etiology of SNN is still uncertain. Sclerotic evolution of small hemangiomas because of the finding of a "feeding vessel" or trauma with or without previous parasitic infestation [7-9] is suggested. In our case the appearance of small vein thrombosis at the borders of the lesion suggested a necrotic process with localized liver tissue infarction. A hypercoagulable state was not confirmed by serum analysis of protein C, S and antithrombin. From a pathological point of view, SNN appears well-delineated by a dense fibrohyalinized fibrotic capsule with a principally necrotic core containing an eosinophil granular matter combined with calcifications, cholesterol, foam cells, and some inflammatory cells. Characteristically Ziehl-Neelsen, Gram and PAS stains do not reveal bacteria or fungi, and the pathogenesis of these cases remains uncertain. The histological findings of our case were in keeping with the above-mentioned features.

Surgical resection is strongly recommended by our team in agreement with the literature [4,10,11] due to the inability to differentiate SNN from malignant lesions.

## Conclusion

SNN of the liver is a lesion without malignant potential. Nevertheless it can mimic HCC, metastasis, abscess or non-specific disease both clinically as well as imaging-wise. Therefore it is important to exclude hepatic malignancy.

Although the behavior of this lesion is benign diagnosis is usually uncertain making surgical resection imperative.

## Consent

Written informed consent was obtained from the patient for publication of this case report and accompanying images. A copy of the written consent is available for review by the Editor-in-Chief of this journal.

## Competing interests

The authors declare that they have no competing interests.

## Authors' contributions

SD, DK, AS and CD analyzed and interpreted the patient data. CT and IP interpreted the computed tomography image. All authors read and approved the final manuscript.
